# Bladder cancer: detection and image quality compared among iShim, RESOLVE, and ss-EPI diffusion-weighted MR imaging with high *b* value at 3.0 T MRI

**DOI:** 10.1097/MD.0000000000009292

**Published:** 2017-12-15

**Authors:** Hongyi Li, Lin Liu, Qinglei Shi, Alto Stemmer, Hong Zeng, Yi Li, Mengchao Zhang

**Affiliations:** a Department of Radiology, China-Japan Union Hospital of Jilin University, Xiantai Changchun, Jilin, China; b MR Scientific Specialist Siemens Healthcare Ltd. Diagnostic Imaging, Wangjing, Zhonghuan, Nanlu, Beijing, China, 100102; c MR Application Predevelopment Siemens Healthcare GmbH, Erlangen, Germany.

**Keywords:** adults, artifacts, limit of detection, MR-diffusion weighted imaging, urogenital neoplasms

## Abstract

To compare the detection of bladder neoplasms and image quality among the diffusion-weighted imaging (DWI) acquired by the prototype single-shot echo-planar-imaging (ss-EPI) sequence for integrated slice-specific dynamic shimming (iShim), readout segmentation of long variable echo trains (RESOLVE) and conventional ss-EPI sequences.

Around 63 patients with 77 bladder lesions were enrolled. The MR protocol included T1WI, T2WI and 3 types of DWI. The sensitivity, specificity, positive predictive value (PPV), negative predictive value (NPV), and accuracy of each DWI for the detection of bladder tumor were computed. The subjective scores of imaging quality, diagnostic confidence, and detection of tumors of stage T2 or greater were recorded. The contrast-to-noise ratio (CNR), signal intensity ratios, and apparent diffusion coefficient (ADC) values were measured. The univariate analysis of variance technique, the Friedman test, and Bland–Altman plots were used in the statistical analysis. Observer performance of tumor T stage was tested using receiver operating characteristic (ROC) curve analysis.

The sensitivity, NPV, and accuracy of iShim (92.75%; 61.54%; 93.51%) for detection of bladder tumor were superior to those of RESOLVE (84.06%; 42.11%; 85.71%) and ss-EPI (86.96%; 47.06%; 88.31%). All qualitative scores of iShim were higher than RESOLVE (all *P* < .05) and ss-EPI (all *P* < .05). The CNR, signal intensity ratios between bladder lesion and urine, lesion, and submucosal stalk (or nearby normal bladder wall), and between distal normal bladder wall and urine of iShim (39.84 ± 12.11, 2.40 ± 0.60, 1.98 ± 0.43, 1.28 ± 0.16) were higher than RESOLVE (16.97 ± 7.08, 1.62 ± 0.41, 1.52 ± 0.42, 1.15 ± 0.29, all *P* < .05) and ss-EPI (27.89 ± 9.65, 1.66 ± 0.46, 1.57 ± 0.50, 0.99 ± 0.22, all *P* < .05). No significant difference of ADC values were found for iShim and RESOLVE (*P*=0.46), iShim, and ss-EPI (*P* = 0.97), RESOLVE and ss-EPI (*P* = .48). The *A*_*z*_ value for the detection of tumors of stage T2 or greater was slightly higher with the iShim DWI sequence (0.89) than with the RESOLVE (0.87, *P* = 0.72) or ss-EPI (0.85, *P* = .38) sequence.

The iShim DWI has relatively better detection of bladder tumor and image quality without significant ADC value difference.

## 1 Introduction

Bladder cancer is one of the most common urinary tract malignancies, causing notable morbidity and mortality.^[1,2]^ Clinical staging of the primary tumor with bimanual examination, cystoscopy, and transurethral resection (TUR) of bladder tumor is associated with an inaccuracy rate from 23% to 50%.^[3–5]^ Therefore, obtaining an accurate imaging study is important to facilitate choosing optimal management methods. Diffusion-weighted imaging (DWI) produced by single-shot echo planar imaging (ss-EPI) has played an important role in the multiparametric MRI and is a useful and reasonably accurate technique to detect and evaluate the extent of bladder cancer.^[6–9]^ However, the limitations of ss-EPI DWI, especially with high b value, including strong magnetic susceptibility artifacts, relatively poor spatial resolution and geometric distortion caused varying the accuracy of detecting and assessing the bladder cancer.^[10–13]^ It is occasionally unable to determinate if the tumor margin is smooth on DWI, which makes the correct staging of the cancer.^[8]^ Moreover, susceptibility artifacts from gas in the small intestine, colon, and rectum around the urinary bladder can potentially cause image distortion with abnormally high signal intensity (SI) of adjacent bladder walls on DWI generated from 3.0 T MRI, that may be misrecognized as flat-type tumor. What is more, bladder tumor less than 1 cm may be missed on DWI.^[9,14–16]^ Therefore, further improvement of the conspicuity of bladder tumor and image quality of DWI were desirable.

Recently, the techniques of readout segmentation of long variable echo trains (RESOLVE) and integrated slice-specific dynamic shimming (iShim) DWI provide better detection and image quality in rectal, prostate, kidney, neck, breast, and the whole body compared with ss-EPI DWI.^[17–22]^ However, there were not any reports concerning applications of iShim and RESOLVE DWI in bladder tumor. Hence, we compared detection of bladder tumor and image quality among iShim, RESOLVE, and ss-EPI sequences at 3.0 T MRI.

## 2 Materials and methods

### 2.1 Patients

This study was approved by the Institutional Review Board, and written informed consent was obtained from all patients, because RESOLVE and iShim were 2 research pulse sequences. From August 2015 to May 2016, 63 patients were collected, who presented with gross (macroscopic) hematuria and who had normal findings from upper urinary tract ultrasonographic evaluation. Exclusion criteria included upper urinary tract tumors or stones, a history of urinary tract trauma, contraindications to MR imaging or cystoscopy and refusal to consent to the study. The 63 patients were examined by using MR imaging and, subsequently conventional cystoscopy within 48 hours. The population included 46 (73.0%) male, 17 (27.0%) female, average age: 63.6 ± 10.6 years, range: 44 to 85 years.

### 2.2 MR examination

All MR examinations were performed on a 3.0 T MR scanner (MAGNETOM Skyra, Siemens AG, Erlangen, Germany). Adequate bladder distention can be achieved by instructing the patient to start drinking an adequate amount of water 30 minutes to 1 hour before the MRI study. Images were acquired in a transverse orientation. T1-weighted, T2-weighted images, and DWI images with approximately same scanning time acquired by 3 sequences (iShim, RESOLVE, and ss-EPI), respectively, were performed in the axial planes with the MR protocol listed in Table 1.

**Table 1:**
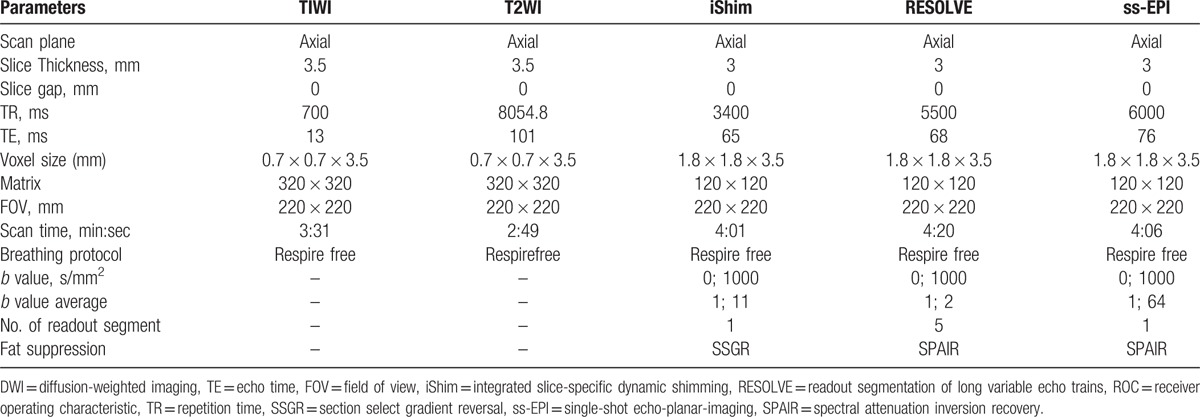
Imaging parameters for T1-and T2-weighted MR imaging and iShim, RESOLVE, and ss-EPI DW imaging sequences.

### 2.3 Image analysis

Images were analyzed by 2 radiologists with 8 and 6 years of abdominal experience, respectively, who were blinded to the results of conventional cystoscopy, read all DWI images in 3 separate sets (iShim, RESOLVE, and ss-EPI) with 2 weeks apart between 2 sets independently. Bladder tumors on DWI images had high SI relative to the bladder wall and surrounding urine. Differences in the assessment were resolved by means of consensus.

### 2.4 Reference standard

Conventional cystoscopy was performed by a urologist with 5 years of experience as a urology consultant who was blinded to the results of MR imaging. In patients treated with radical cystectomy (n = 20), tumor appearance and size were established from the final histologic report; otherwise, tumor appearance and size were established at cystoscopy by using a ureteric catheter. Morphology was classified as fungating (overlying mucosa of the tumor was ulcerated, with fungation into the bladder lumen), nodular (intact mucosa overlying the tumor), or papillary (looked like a papilla).^[14]^

### 2.5 Qualitative analysis of imaging quality

At the same time of reading the characteristics of bladder tumors on 3 DWIs, the 2 radiologists independently graded the qualitative score according to the below items. The depiction of the bladder lesion edge and normal bladder wall with a 5-point scales: 1 = indicated unacceptable depiction; 2 = poor and severely blurred depiction; 3 = moderate depiction; 4 = clear depiction with slight blurring; and 5 = excellent depiction with no blurring; The magnetic susceptibility artifacts and the flow artifacts of urine were also assessed: 1 = severe artifacts; 2 = moderate artifacts; 3 = mild artifacts; 4 = minimal artifacts; 5 = no artifacts. The confidence scores of the DW imaging quality which can guarantee the bladder tumor staging: 1 = limited, 2 = acceptable, 3 = confident.

Each radiologist also assigned a confidence level for the presence of a tumor of stage T2 or greater using a 4-point scale: 1, definitely absent; 2, possibly absent; 3, possibly present; or 4, definitely present. Two radiologists who were blinded to the pathological results read all DWI images in 3 separate sets (iShim, RESOLVE, and ss-EPI) with 1 week apart between 2 sets independently and differences in the assessment were resolved by means of consensus. We divided stage T1 tumors from T2 or greater tumors because the preoperative T stage was a determinant of treatment options: TUR is often chosen for stage T1 tumors, but cystectomy is chosen for tumors of stage T2 or greater.

### 2.6 Quantitative analysis of imaging quality

Quantitative image analysis was performed by one of the abovementioned abdominal radiologists. The radiologist drawn regions of interest (ROIs) to measure the signal intensity of the bladder cancer (SI_BCA_), the submucosal stalk or normal bladder wall nearby the lesion (SI_nearby_), the distal normal bladder wall (SI_distal__wall_), the urine (SI_urine_), and the standard deviation (SD) of the background signal intensity. ROIs for SI_BCA_ were manually drawn with the maximum diameter of the lesion and placed inside the inner edge of the lesion, avoiding partial volume effects. All ROIs were put on the same slice on which the maximum area of the bladder cancer shown. The ROI area of measuring the SI_urine_ and the SD of the background signal intensity was100 mm^2^. Contrast-to-noise ratio for lesion (CNR) were defined by the following relationships: CNR = (SI_BCA_− SI_distal__wall_)/SD. Further, the signal intensity ratios were calculated by the following formulas: Ratio_L/U_ = SI_BCA_/SI_urine_; Ratio_L/N_ = SI_BCA_/SI_nearby_; Ratio_L/D_ = SI_BCA_/SI_distal__wall_; Ratio_D/U_ = SI_distal__wall_/SI_urine_.

### 2.7 Measurement of apparent diffusion coefficient (ADC)

The ADCs of bladder tumors were measured by the same method to the signal intensity at the same time. The same radiologist drew ROIs on the ADC maps.

### 2.8 Statistical analysis

Statistical analyses were performed using commercially available software SPSS 18.0 (SPSS, Chicago, IL), with conventional cystoscopy or the final histopathologic report as the reference standard. We evaluated the sensitivity, specificity, positive predictive value (PPV), negative predictive value (NPV), and accuracy of 3 kinds of DWI for detecting bladder tumors. In addition detection of bladder tumors by site on DW with conventional cystoscopy as the reference standard was evaluated according to the size, location, and morphology of the bladder tumors.

To evaluate the performance and agreement of the 2 reviewers at identifying bladder tumors, we applied the statistic. A κ of <0.20 was considered poor; 0.21–0.40, fair; 0.41–0.60, moderate; 0.61–0.80, good; and 0.81–1.00, excellent. A comparison of imaging findings for detecting bladder tumors with the results of cystoscopy and histologic examination was subsequently performed by using the McNemar test. The subjective evaluation scores of iShim, RESOLVE, and ss-EPI DWI were compared using Friedman test. Univariate analysis of variance or Friedman test was applied for CNR, signal intensity ratio, and ADC. Univariate analysis of variance was applied for group comparison with LSD correction. To further verify the concordance of ADC values between iShim, RESOLVE, and ss-EPI, Bland–Altman plots were created.

Diagnostic accuracy of staging with DWI MR images as compared with pathologic stage was assessed. Differences in sensitivity, specificity, and diagnostic accuracy for each image set were evaluated. Receiver operating characteristic (ROC) curves were fit to the radiologists’ confidence rating using software MedCalc for Windows (version 12.7; MedCalc Software, Ostend, Belgium). Observer performance for each sequence was estimated by calculating the area under the ROC curve (*A*_z_). Differences between *A*_*z*_ values were estimated. A 2-tailed *P* value < .05 was considered statistically significant difference.

## 3 Results

### 3.1 Lesion characteristics

Sixty-three patients had a total of 77 lesions and 55 patients were diagnosed with bladder tumors (45 patients with single lesion and 10 patients with multiple lessions). Bladder tumors were diagnosed in 69 (89.61%) of the 77 lesions; cystitis, in 3 (3.90%); benign prostatic hyperplasia, in 3 (3.90%); and ureteric tumor in 2 (2.59%). Bladder tumor size ranged from 0.21 to 5.53 cm (mean: 1.96 cm ± 3.63 cm). The locations of the 69 bladder tumors included the right lateral wall (n = 15), left lateral wall (n = 11), anterior wall (n = 8), posterior wall (n = 26), neck (n = 4), and dome (n = 5). The shape of bladder tumor was fungating in 24 (37.78%) of the 69 lesions, nodular in 30 (43.48%), and papillary in 15 (21.74%).

The histologic diagnosis was transitional cell carcinoma in 61 (88.41%) of the 69 lesions with tumors and squamous cell carcinoma in 8 (11.59%). Muscle invasion by the tumor cells was present in 20 lesions (28.99%), while the remaining 49 lesions (71.01%) confirmed to be superficial ones.

Tumor extension was pathologically proven for stage T1 in 25 (45.46%) patients, T2 in 20 (36.36%), T3 in 8 (14.55%), and T4 (3.63%) in two.

### 3.2 Detection of bladder tumors

**Figure 1:**
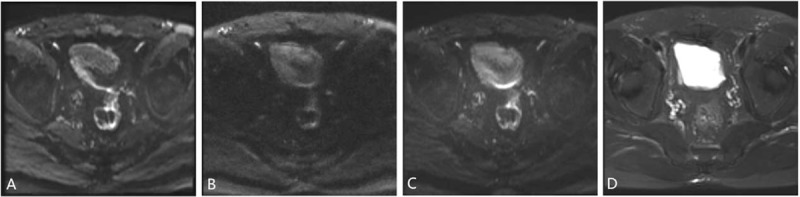
A 58-year-old male with no bladder lesion. The gas contained in rectum can be well recognized on (D) T2WI. On (A) iShim and (C) ss-EPI, the posterior bladder wall had abnormally high SI caused by susceptibility artifacts and can be misrecognized as a flat-type cancer and (B) RESOLVE showed no high SI. The artifacts due to gas in rectum can be well recognized on (D) T2WI. iShim = integrated slice-specific dynamic shimming, RESOLVE = readout segmentation of long variable echo trains, ss-EPI = single-shot echo-planar-imaging.

**Figure 2:**
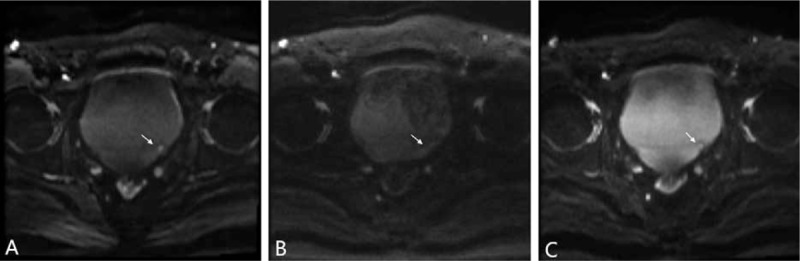
Comparison of (A) iShim, (B) RESOLVE and (C) ss-EPI DWI images (b = 1000 sec/mm^2^) in a 50-year-old male patient with single bladder cancer located in posterior wall (arrow). The size of the lesion is 0.4 cm, which is more clearly visible on (A) iShim DWI compare with (B) RESOLVE and (C) ss-EPI. Confidence score on (A) iShim, (B) RESOLVE and (C) ss-EPI is 3,1 and 2. DWI = diffusion-weighted imaging, iShim = integrated slice-specific dynamic shimming, RESOLVE = readout segmentation of long variable echo trains, ss-EPI = single-shot echo-planar-imaging.

**Figure 3:**
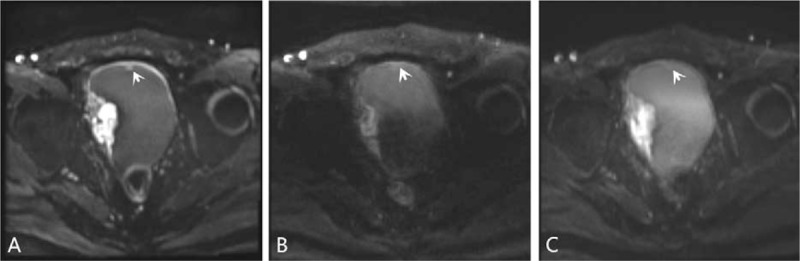
Comparison of (A) iShim, (B) RESOLVE, and (C) ss-EPI DWI (*b* = 1000 s/mm^2^) in a 66-year-old male patient with multiple bladder cancers. The smaller lesion located in anterior wall (size = 0.5 cm) (arrow) is clearly seen on (A) iShim. The same lesion could not be seen on (B) RESOLVE and the depiction of lesion is poor on (C) ss-EPI. Confidence score for smaller lesion on (A) iShim, (B) RESOLVE, and (C) ss-EPI is 3, 1, and 2. Images acquired with (B) RESOLVE and (C) ss-EPI DWI depict larger bladder cancer less clearly than in (A) iShim because of flow artifacts. The edge of lesion and normal bladder wall can be seen more clearly on (A) iShim DWI. Confidence score for larger lesion on (A) iShim, (B) RESOLVE, and (C) ss-EPI is 3, 2 and 2. iShim = integrated slice-specific dynamic shimming, RESOLVE = readout segmentation of long variable echo trains, ss-EPI = single-shot echo-planar-imaging.

Sensitivity, specificity, PPV, NPV, and accuracy of 3 DWIs for the detection of bladder tumors for the consensus of the 2 evaluators are summarized in Table 2. iShim, RESOLVE, and ss-EPI DWI enabled detection of 64, 58, and 60 of the 69 tumors noted at conventional cystoscopy, with 3, 2, and 3 false-positive findings caused by susceptibility artifacts (Fig. 1). The number of bladder tumors on iShim, RESOLVE, and ss-EPI DWI according to the size, shape, and location of bladder tumor noted at conventional cystoscopy are listed in Table 3. Of the 24 bladder tumors confirmed by histologic reports or conventional cystoscopy smaller than 1 cm (range: 0.21–0.93 cm), 4 (16.67%) were not detected on iShim; 10 (41.67%) were not detected on RESOLVE; and 8 (33.33%) were not detected on ss-EPI. An example of the advantages of iShim DWI detecting small lesions was presented in Figures 2 and 3.

**Table 2:**
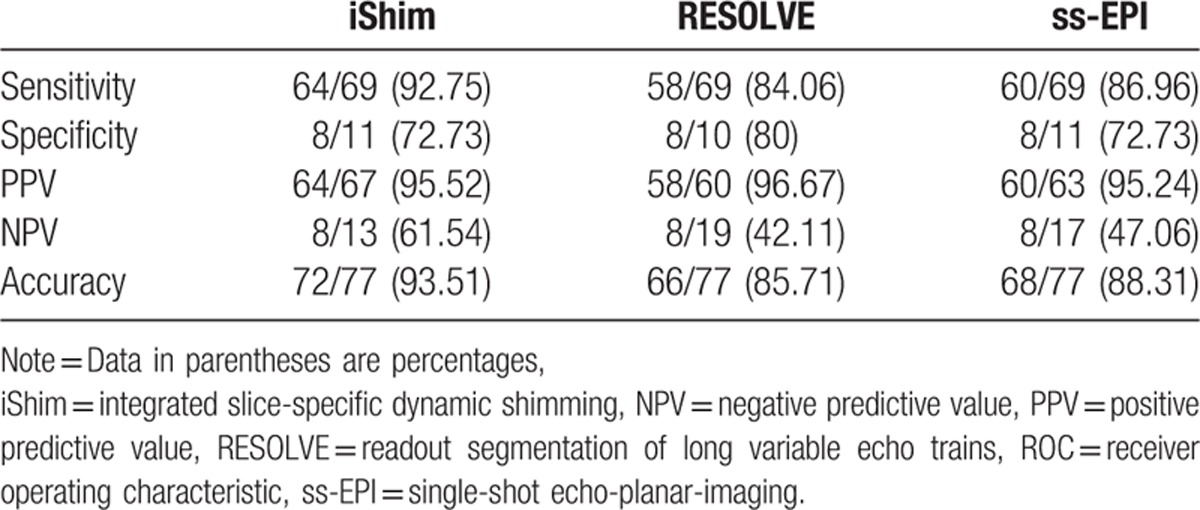
Comparisons among iShim, RESOLVE, and ss-EPI for detection of bladder tumors.

**Table 3:**
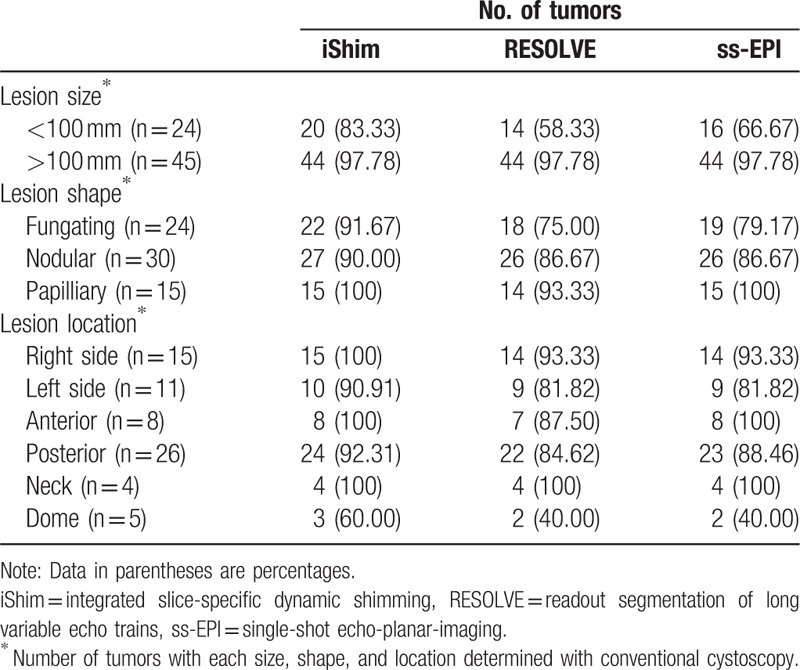
Detection of bladder tumors by size, shape and locations on iShim, RESOLVE, and ss-EPI with conventional cystoscopy as the reference standard.

The reviewers’ interpretations of iShim agreed with the findings at conventional cystoscopy for 64 malignant bladder tumors. The agreement between iShim, RESOLVE, ss-EPI and conventional cystoscopic findings or the identification of bladder tumors were good and moderate (κ=0.73; 0.52; 0.58). By using the McNemar test, no significant difference between iShim DWI and cystoscopy (*P* = .06). A significant difference was found between RESOLVE, ss-EPI, and cystoscopy (*P* < .01; *P* = .04).

### 3.3 Qualitative analysis of imaging quality

**Figure 4:**
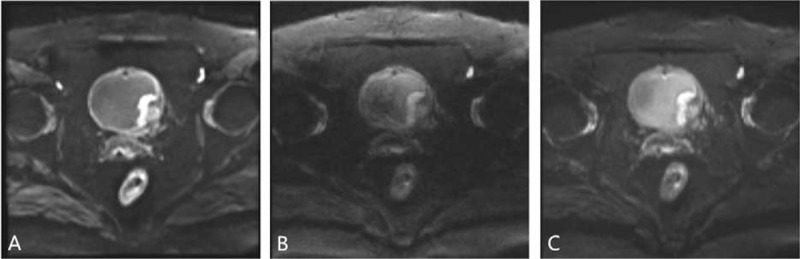
Comparison of (A) iShim, (B) RESOLVE, and (C) ss-EPI DWI (b = 1000 sec/mm^2^) in a 67-year-old female patient with bladder cancer. The lesion especially the edge of lesion and normal bladder wall can be seen more clearly on (A) iShim DWI. On (B) RESOLVE and (C) ss-EPI, the lesion edge and bladder wall obscured by image artifacts. Confidence score on (A) iShim, (B) RESOLVE, and (C) ss-EPI is 3, 3 and 3. DWI = diffusion-weighted imaging, iShim = integrated slice-specific dynamic shimming, RESOLVE = readout segmentation of long variable echo trains, ss-EPI = single-shot echo-planar-imaging.

Interobserver agreement of the depiction of the bladder lesion edge and normal bladder wall, image artifacts, and confidence scores of staging the bladder tumor were good or excellent in all 3 DWI sequences (iShim: 0.89, 0.86, 0.78; RESOLVE: 0.81, 0.71, 0.73; ss-EPI: 0.76, 0.85, 0.75). The subjective evaluation scores of both evaluators in Table 4 shows that the depiction of the bladder lesion edge and normal bladder wall, SI and flow artifacts of urine and confidence scores in iShim sequence were better than in RESOLVE and ss-EPI. Examples of the advantages of iShim DWI are presented in Figures 2 and 4.

**Table 4:**
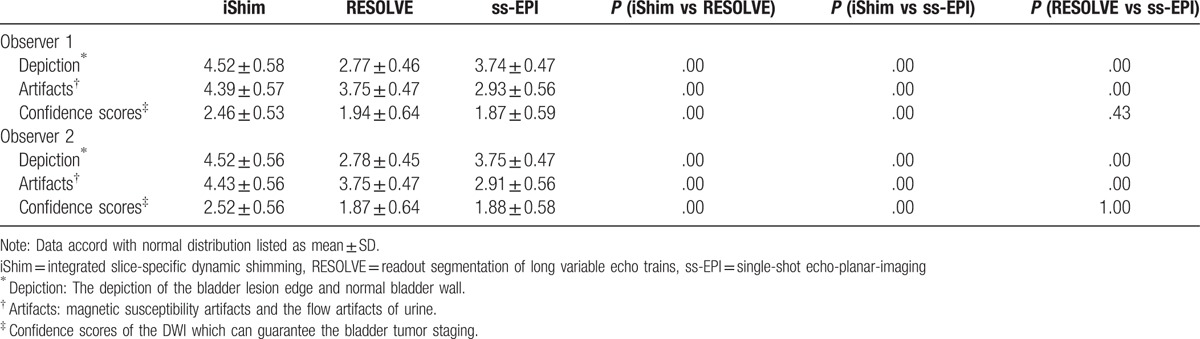
Comparisons of qualitative scores and confidence scores among iShim, RESOLVE, and ss-EPI.

### 3.4 Quantitative analysis of imaging quality

The mean and standard deviation of CNR and all signal intensity ratios were listed in Table 5. The CNR of iShim were significantly better than RESOLVE and ss-EPI (*P* < 0.01). Better Ratio_L/U_, Ratio_L/S_, and Ratio_D/U_ were found in iShim than in RESOLVE (*P* < .01; *P* < .01; *P* = .01), and in ss-EPI (*P* < 0.01; *P* < 0.01; *P* < .01).

**Table 5:**
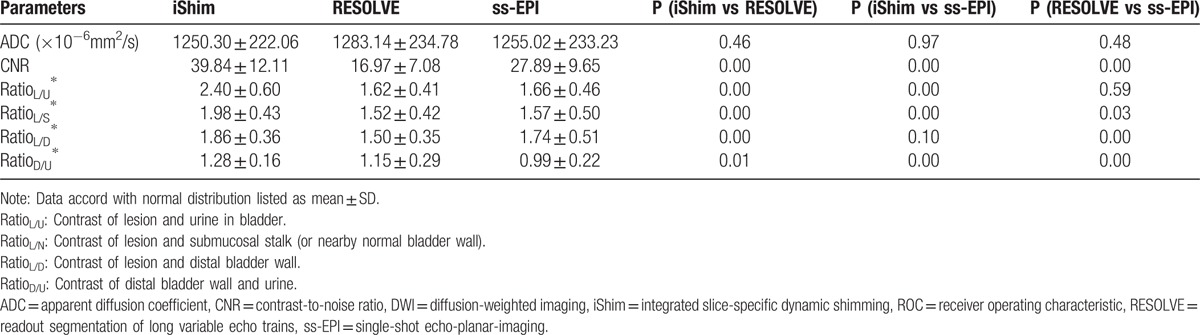
Comparison of quantitative analysis of imaging quality among iShim, RESOLVE, and ss-EPI DWI sequences.

**Figure 5:**
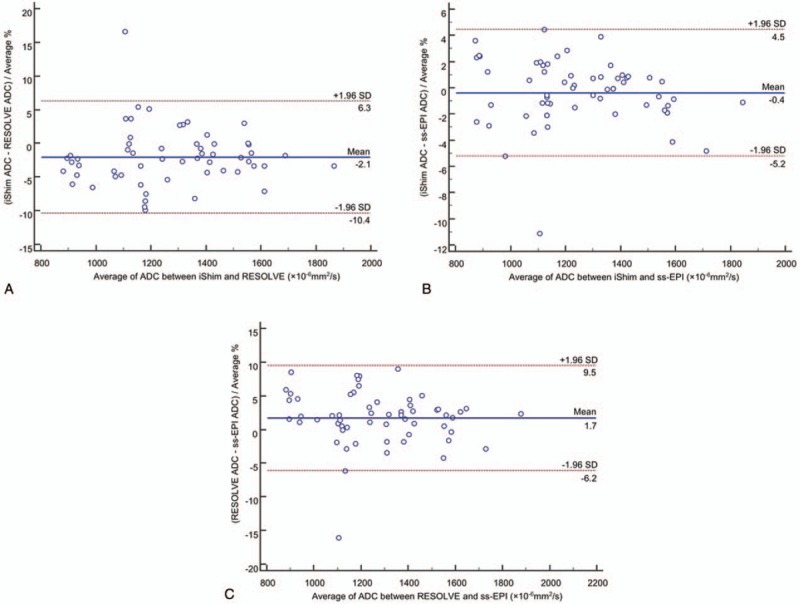
Bland–Altman plots for the quantitative ADC values: (A) ADC values between iShim and RESOLVE; (B) ADC values between iShim and ss-EPI; (c) ADC values between RESOLVE and ss-EPI. The mean ADC difference between two sequences and the limits of agreement (±1.96 times the standard deviation[SD]) are displayed. ADC = apparent diffusion coefficient, iShim = integrated slice-specific dynamic shimming, RESOLVE = readout segmentation of long variable echo trains, ss-EPI = single-shot echo-planar-imaging.

The mean and standard deviation of ADC of iShim, RESOLVE, and ss-EPI are listed in Table 5. There were not any significant differences between the ADC value for iShim and RESOLVE (*P* = .46), iShim, and ss-EPI (*P* = .97), RESOLVE and ss-EPI (*P* = .48). Bland–Altman plots demonstrated that the mean difference in percentage and limits of agreement in ADC between iShim and RESOLVE, iShim and ss-EPI, and iShim and ss-EPI were: −2.1% (95%CI: −10.4–6.3%); −0.4% (95%CI: −5.2–4.5%), and 1.7% (95%CI: −6.2–9.5%) (Fig. 5). There was only one pair of samples beyond the 95% limits of agreements, which indicating a respectively high concordance of analysis between iShim, RESOLVE, and ss-EPI.

### 3.5 T Stage of bladder tumors

The sensitivities, specificities, accuracies, and *A*_*z*_ values for the detection of urinary bladder tumors of stage T2 or greater are summarized in Table 6.

**Table 6:**
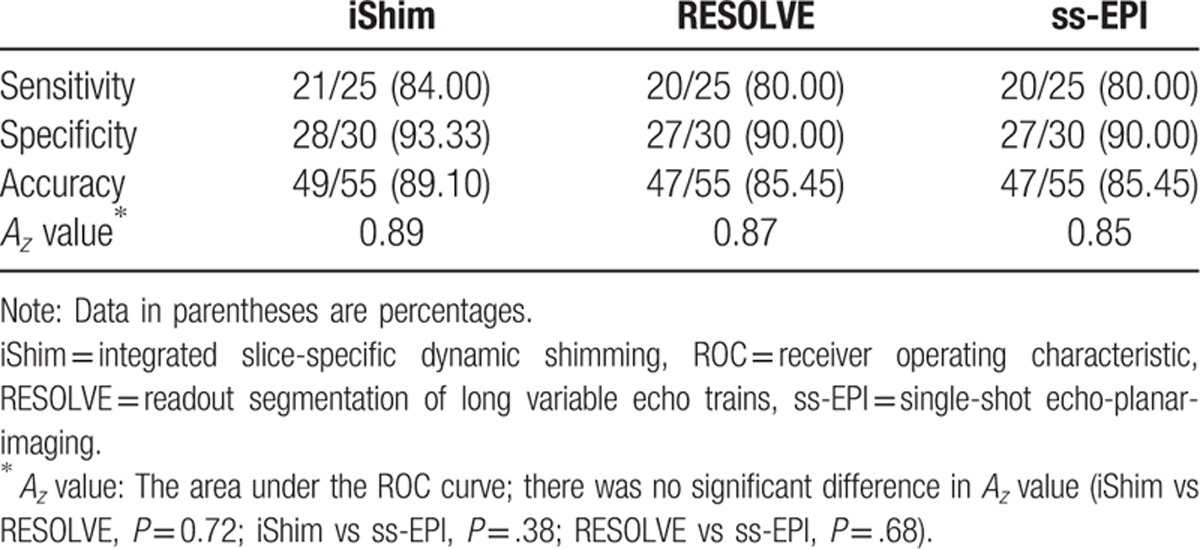
Sensitivity, specificity, accuracy, and area under the ROC curve (*A*_*z*_) for tumors of stage T2 or greater.

The sensitivity and specificity were higher with iShim (84.00%; 93.33%) than with RESOLVE or ss-EPI (80.00%; 90.00% each). The accuracy was identical (85.45% each) between RESOLVE and ss-EPI, which was lower than iShim sequence (89.10%). The *A*_*z*_ value for the detection of tumors of stage T2 or greater was slightly higher with the iShim DWI sequence (0.89) than with the RESOLVE (0.87, *P* = .72) or ss-EPI (0.85, *P* = 0.38) sequence.

## 4 Discussion

In this study, we compared detection of the bladder tumor and image quality among iShim, RESOLVE, and ss-EPI DWI with high *b* value at 3.0 T MRI.

As we know, the imaging quality is a key point of detecting and evaluating a lesion. In previous studies, because of poor spatial resolution and insufficiency of tissue contrast between small tumors and surrounding structure, bladder tumor <1 cm may be missed on ss-EPI DWI.^[9,14–16]^ In this study, the higher CNR and signal ratios between the bladder tumor, normal bladder wall, and the urine were shown on the iShim DWI. In addition, iShim had better performance of subjective scores. The clearer border and shape of the edge provided by iShim DWI let the readers have more confidence to stage the tumor (Figs. 2 and 3). Previous studies also showed the similar results.^[20–22]^ The superior performance of the iShim sequence compared with RESOLVE and ss-EPI can be attributed to the procedure of dynamic frequency adjustment and slice-selective shimming that reduces field inhomogeneities (ΔB0) and thus related artifacts.^[20,21,23]^ As a result, iShim had higher sensitivity, NPV, and accuracy of detecting the bladder tumor than RESOLVE and ss-EPI. According to the further analysis based on the size and shape found at conventional cystoscopy, we got a result that more bladder tumors, especially the small bladder tumors (<1 cm) with fungating and nodular shape were observed using iShim DWI. On the ss-EPI DWI, urine showed mild high SI in patients with bladder cancer due to the impaction of T2 component on DWI, which lead to underdetection of intraluminal tumor.^[24]^ We found that iShim DWI significantly prevented the high signal of urine and eliminated the flow artifacts of urine (Figs. 2 and 4).

RESOLVE DWI has better performance of decreasing magnetic susceptibility artifacts, which can cause false high SI of adjacent bladder walls, compared to iShim and ss-EPI (Fig. 1). Contrary to other studies,^[18,19,25]^ the RESOLVE DWI has the poorer performance of detection of the bladder tumor and imaging quality compared to other sequences. Although spatial distortion is reduced as readout-time and thus accumulation of phase errors is reduced by segmenting echo train in frequency-encoding direction, the longer scanning time is required in RESOLVE comparing with other DWIs.^[13,26]^ This is not beneficial to patients with bladder cancer, especially for those with multiple lesions because it is difficult for patients to hold a full bladder for a long time. Therefore, we think that selecting the RESLOVE sequence for DW imaging of the bladder tumor is not suitable.

In addition, for detecting the tumor in different locations of the bladder, all of the 3 DWI sequences had no performance. The reason may be that the loss of normal signals of the thin muscle layer at some locations of the bladder wall, such as the junction of the lateroposterior wall on the axial DWI.

Another important parameter of DWI is ADC value. ADC value could be a better biomarker predicting histopathologic grading, aggressiveness, and tumor response to chemoradiation therapy.^[8,27–30]^ Thus, we think that it is necessary to identify whether there are differences of ADC value among 3 kinds of DWI sequences. In this study, the ADC values produced by the iShim, RESLOVE, and ss-EPI DWI were consistent. This was in good agreement with previous studies.^[20,31]^

We also differentiated bladder tumors between T1 or lower and T2 or higher because the treatment options differ considerably. Although a statistically signifcant for the detection of tumors of stage T2 or greater difference was not found among 3 DWIs, a potentially clinically signifcant difference was found: the sensitivity, specifcity, and accuracy were higher in iShim DWI, which provided information that reduced understaging or overstaging comparing with RESOLVE and ss-EPI. In addition, the higher detection ratio of bladder lessions, especially of small lesion (<1 cm) and better image quality using iShim were found comparing with other 2 DWIs with approximately same scanning time. Therefore, DW imaging based on iShim is a clinically promising technique to improve the detection and image quality for the purpose of evaluating lesions in patients with bladder tumors.

This study had some limitations. First, we did not assess the histologic grades of urothelial carcinomas because our purpose was to evaluate the detection of bladder tumor and image quality of 3 DWIs. However, further study is needed to assess whether the use of iShim DWI could improve such diagnose. Second, in this study, the comparison was not applied for scars and reactive tissue after chemotherapy and radiation therapy. Third, metal prostheses or implants were not adequately examined in this study.

In conclusion, iShim has relatively better detection of the bladder tumor and image quality among 3 sequences of DWI without significant ADC value difference.
